# Specific Instability of HLA-A*03:01 Expression in HEK-293 Cells

**DOI:** 10.3390/ijms262311357

**Published:** 2025-11-24

**Authors:** María Area-Navarro, Alba Pastor-Moreno, Erika Scholz, Américo Cerqueira, Adrián Tirado-Herranz, Miguel Marcilla, Francesc Canals, Manel Juan, José Ramón Palacio, Iñaki Alvarez

**Affiliations:** 1 Department of Cell Biology, Physiology and Immunology, Institute of Biotechnology and Biomedicine, Autonomous University of Barcelona, 08193 Bellaterra, Spain; maria.area@uab.cat (M.A.-N.); alba.pastor@uab.cat (A.P.-M.); erika.scholz@iispv.cat (E.S.); adrian.tirado.herranz@gmail.com (A.T.-H.); joseramon.palacio@uab.cat (J.R.P.); 2 Proteomics Unit, Spanish National Biotechnology Centre, 28049 Madrid, Spain; americo.cerqueira@estudiante.uam.es (A.C.); mmarcilla@cnb.csic.es (M.M.); 3 Proteomics Laboratory, Vall d’Hebron Institute of Oncology (VHIO), 08035 Barcelona, Spain; fcanals@vhio.net; 4 Department of Immunology, Hospital Clínic de Barcelona, 08036 Barcelona, Spain; mjuan@clinic.cat

**Keywords:** HLA, antigen presentation, immunopeptidome, HEK-293, peptides

## Abstract

HEK-293 is a highly transfectable human cell line widely used as a model for protein expression. Since large amounts of cells are often required for the purification of HLA immunopeptidomes, suspension-growing variants, such as HEK-293F, facilitate the generation of sufficient cell quantities. The HLA class I-typing of these cells is HLA-A*02:01, -A*03:01, -B*07:02, and -C*07:02. HEK-293T cells have been previously used as a source of HLA peptide ligands derived from SARS-CoV-2 proteins. In this study, we purified and analyzed the HLA-I immunopeptidome of HEK-293 and HEK-293F cells using mass spectrometry. Cell surface expression of specific HLA-I allotypes was determined using flow cytometry with allele-specific antibodies. The HLA-I immunopeptidome of HEK-293 cells contained ligands from all three HLA-I allotypes, whereas that of HEK-293F cells lacked peptides derived from HLA-A*03:01. Flow cytometry experiments confirmed the absence of HLA-A*03:01 expression on the surface of HEK-293F cells. Additionally, we generated a HEK-293 transfectant co-expressing the β5i proteasome subunit and the SARS-CoV-2 Spike protein. This transfectant showed selective loss of HLA-A*03:01 expression, suggesting that HEK-293 linages tend to specifically lose this allotype. We propose that HEK-293F cells are unsuitable for the identification of HLA-A*03:01 ligands or for stimulating T-cell responses restricted to this allele. Moreover, HLA-A*03:01 expression should be regularly monitored in HEK-293-derived cells.

## 1. Introduction

The study of the class I Human Leukocyte Antigen (HLA-I) immunopeptidome has become an area of great interest across multiple fields of immunology. It plays a role in the identification of T-cell epitopes derived from pathogens, neoantigens, and tumor-associated targets relevant to cancer immunotherapy, as well as autoantigen targets implicated in autoimmunity. In addition, alterations in the HLA immunopeptidome can reflect modifications in the antigen processing pathways [1].

The Human Embryonic Kidney (HEK)-293 cell line is widely used for the study of several cellular processes due to its high transfectability and its ability to express functional proteins from diverse tissues, despite its epithelial origin. It was originally established in 1977 through the transformation of human embryonic kidney cells with adenovirus 5 [2,3,4]. Several derivatives of the HEK-293 cell line have been developed for different applications. For instance, HEK-293T and HEK-293E were generated to enhance recombinant protein production for therapeutical purposes. In addition, suspension-adapted variants such as HEK-293F and HEK-293H have been established to facilitate large-scale culture and protein expression [5]. The HEK-293 genome is in constant evolution because of chromosomic translocation events that alter copy numbers, suggesting that subcloning and prolonged culture may lead to karyotypic derive. Although all variants originate from the parental HEK-293 cell line, genomic and transcriptomic differences have been reported because of its genome instability. These changes are particularly evidenced in suspension-growing variants [6].

Although recent advances in immunopeptidomics have reduced the number of cells required for analysis, the detection of low-abundance specific peptides often still demands large cell quantities. Thus, suspension-growing cells, such as HEK-293F, enable the rapid generation of high cell densities, facilitating large-scale peptide isolation. Therefore, HEK-293F cells may serve as a suitable cell model for identifying new peptides bound to HLA-A*02:01, HLA-A*03:01, HLA-B*07:02, and HLA-C*07:02, as it is expected that increasing the number of cells enhances the likelihood of detecting less abundant peptides. The HEK-293T cell line has been widely employed for protein production, similar to the parental HEK-293 cell line. During the SARS-CoV-2 pandemic, the HEK-293T cell line was used as a source of peptide–HLA complexes (pHLA) for the purification of the corresponding immunopeptidomes and the identification of SARS-CoV-2-derived HLA-I ligands from infected cells [7,8,9].

In this work, we evaluated whether the HEK-293F cell line could serve as an equivalent model to the HEK-293 cell line in terms of antigen presentation. Analysis of HLA-I immunopeptidomes revealed that the HEK-293F immunopeptidome lacked HLA-A*03:01 ligands, while peptides corresponding to HLA-A*02:01, HLA-B*07:02, and HLA-C*07:02 were identified. In contrast, the HEK-293 HLA-I immunopeptidome contained ligands derived from all four allotypes. Flow cytometry analysis further demonstrated that HEK-293F cells did not express HLA-A*03:01 on the cell surface, while HEK-293 cells expressed all HLA-I molecules. Together, these findings confirm that HEK-293F cells cannot be used as equivalent to HEK-293 cells with respect to antigen presentation.

Our group is interested in identifying SARS-CoV-2-derived peptides presented by cells expressing different proteasomes. To this end, several transfectants have been generated, including one expressing only the type I intermediate proteasome, characterized by the catalytic subunits β1, β2, and β5i. This β5i^+^ cell line was subsequently transfected with the gene encoding the SARS-CoV-2 Spike Omicron variant. Analysis of its HLA-I immunopeptidome revealed the absence of HLA-A*03:01-resctricted peptide ligands. Consistently, flow cytometry analysis confirmed that this transfectant cell line also lacked surface expression of HLA-A*03:01.

These data strongly suggest that HEK-293 cells have an inherent tendency to specifically lose HLA-A*03:01 expression. Consequently, HEK-293F cells cannot be considered equivalent to HEK-293 cells in terms of antigen presentation. Therefore, we recommend that HLA-A*03:01 expression must be regularly evaluated in HEK-293-derived cells when they are employed as antigen-presenting cells.

## 2. Results

### 2.1. Analysis of HEK-293 and HEK-293F Immunopeptidomes

Genomic DNA was extracted from HEK-293 and HEK-293F cells and used for high-resolution HLA class I typing. Both cell lines contained HLA-A*02:01, A*03:01, B*07:02, and C*07:02.

**Figure 1:**
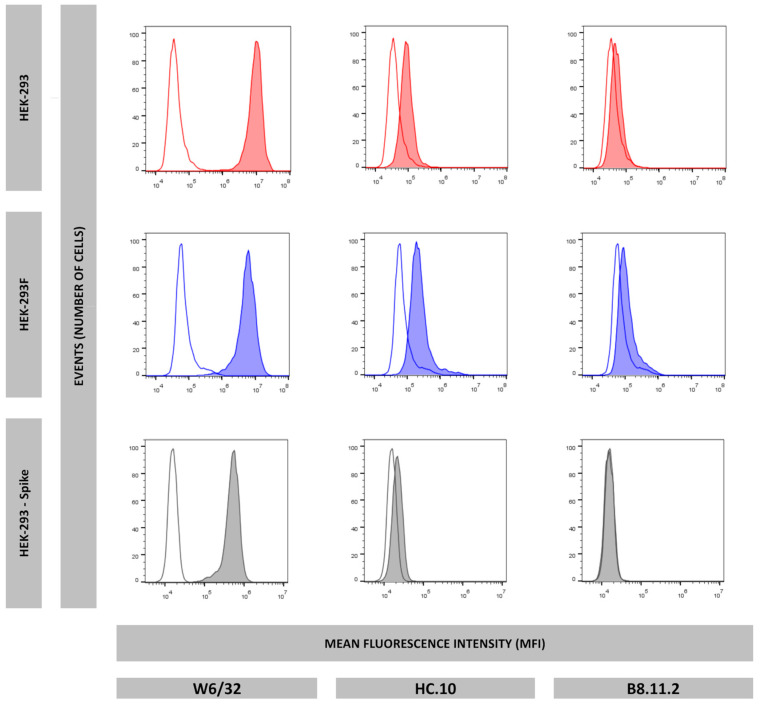
Surface expression of HLA-I well-conformed molecules (W6/32), HLA-I empty molecules (HC.10), and HLA-DR molecules (B8.11.2) of HEK-293 (red), HEK-293F (blue), and HEK-293-Spike (grey). Non-colored histograms represent second antibody control.

To confirm whether the HEK-293F cell line could be employed as a cellular model for antigen presentation of the HLA-I immunopeptidomes comparable to HEK-293, flow cytometry experiments were performed to evaluate the surface expression of HLA-I molecules in HEK-293 and HEK-293F. Monoclonal antibodies W6/32, HC-10, and B8.11.2 were used to detect total well-conformed HLA-I molecules, empty HLA-I molecules, and HLA-DR, respectively. Staining with W6/32 was positive for both cell lines, slightly positive with HC-10, and negative with B8.11.2 (Figure 1). These results indicate that HEK-293 and HEK-293F cells express HLA-I molecules on the cell surface, mostly stabilized with high-affinity peptides.

HLA-I immunopeptidomes were purified from HEK-293F cells. Approximately 5 × 10^8^ cells were lysed in the presence of Igepal CA-630, and peptide–HLA-I complexes were isolated with immunoaffinity chromatography using the mAb W6/32. Peptides were eluted under acid conditions, fractionated using RP-HPLC, and fragmented using ion trap mass spectrometry.

**Figure 2:**
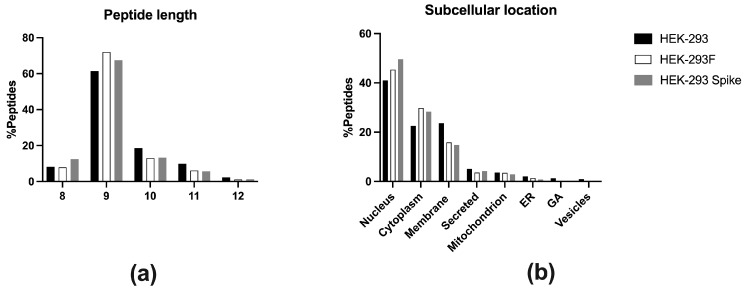
(**a**) Peptide length distribution of the class I immunopeptidome eluted from HEK-293 (black, n = 4133), HEK-293F (white, n = 3085), and HEK-293-Spike (grey, n = 967). Peptides are classified according to 8-mer, 9-mer, 10-mer, 11-mer, and 12-mer. (**b**) Subcellular location of the parental proteins of the eluted peptides from HEK-293 (black, n = 2647), HEK-293F (white, n = 2134), and HEK-293-Spike (grey, n = 800).

A total of 3152 peptides were identified at 1% FDR at peptide level (Supplementary Table S1). Only HLA-I peptide ligands from 8 to 12 residues were considered, and post-translational modifications were not accepted. Thus, in case a peptide was found both in non-modified and modified versions (oxidized, etc.), it was counted as a unique peptide. Using this criterium, 3085 unique peptides were identified. Size distribution of the immunopeptidome is shown in Figure 2a. As expected, most peptide ligands were nonamers (71.9%), followed by peptides of 10 (12.9%), 8 (8.0%), and 11 residues (6.0%). Some 12-mer (1.2%) were also detected. Peptides were derived from 2134 proteins, mostly located in the nucleus (45.4%) and cytosol (30.0%), with peptides also originating from proteins in the plasma membrane (16.0%), secreted (3.6%), mitochondria (3.5%), endoplasmic reticulum (1.4%), vesicles (0.2%), and Golgi apparatus (0.2%) (Figure 2b).

**Figure 3:**
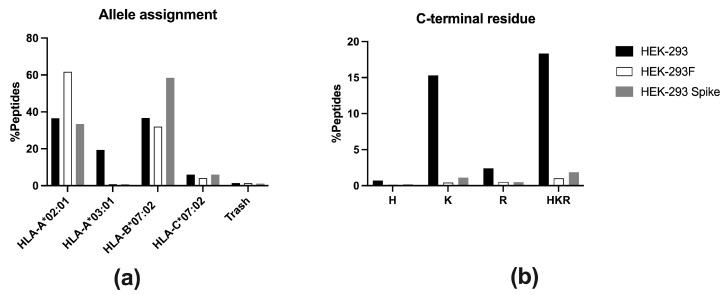
(**a**) Allele assignment of the peptides eluted for HEK-293 (black, n = 4133), HEK-293F (white, n = 3085), and HEK-293-Spike (grey, n = 967) according to MHCMotifDecon-1.0 software. “Trash” refers to peptides with a %Rank ≥ 20 for any HLA-I molecule. The assignment is performed to HLA-A*02:01, -A*03:01, -B*07:02, and -C*07:02. (**b**) Peptides from the HLA-I immunopeptidomes from HEK-293 (black), HEK-293F (white), and HEK-293-Spike (grey) containing basic residues at the C-terminal (PΩ) position. One-letter codes for amino acids are used: H (histidine), K (lysine), and R (arginine).

MHCMotifDecon-1.0 software was used to identify the HLA-I molecule to which each peptide is predicted to bind. It assigned 1904 (61.7%) peptides as ligands of HLA-A*02:01, 986 (32.0%) peptides bound to HLA-B*07:02, and 127 (4.1%) to HLA-C*07:02 (Figure 3a). Forty-three (1.4%) peptides were considered as trash and not assigned to any allotype. Surprisingly, only 25 (0.8%) peptides were predicted to be HLA-A*03:01 ligands (Figure 3a). Some of these were probably contaminants, as their theoretical binding affinity to any allotype was low. The vast majority of peptides bound to HLA-A*03:01 consistently feature basic residues at the C-terminal position (PΩ). Only 1.0% of total peptides contained basic residues in that anchor position (Figure 3b), confirming that HLA-A*03:01 peptide ligands were almost absent in the repertoire.

The absence of HLA-A*03:01 peptide ligands was not expected. To confirm that it was specific of HEK-293F cells, peptides were isolated from a similar number of HEK-293 cells and analyzed using mass spectrometry. A total of 4492 peptides were identified at a 1% FDR at the peptide level (Supplementary Table S2). Following the same criteria as for HEK-293F, a total of 4133 unique peptides were identified.

Most peptide ligands were nonamers (61.2%), followed by peptides of 10 (18.6%), 11 residues (9.8%) and 8-mer (8.2%). Finally, some peptides of 12 residues (2.2%) were also detected (Figure 2a).

Peptides derived from 2647 proteins located in the nucleus (41.0%), plasma membrane (23.7%), cytoplasm (22.5%), secreted (5.1%), mitochondrion (3.6%), endoplasmic reticulum (2.0%), vesicles (0.9%), and Golgi apparatus (1.3%) (Figure 2b).

A similar analysis was performed on the HLA-I immunopeptidome eluted from HEK-293 cells, as previously done for HEK-293F cells, using the MHCMotifDecon-1.0 software. A total of 1510 (36.5%) peptides were assigned as HLA-A*02:01 ligands, 1516 (36.7%) as -B*07:02 ligands, 247 (6.0%) as -C*07:02 ligands, and 58 (1.4%) as trash (Figure 3a). In addition, 801 (19.4%) of the HLA-I ligands were attributed to HLA-A*03:01 (Figure 3a). Thus, HEK-293 cells possess a substantial number of HLA-A*03:01 peptide ligands within their HLA-I immunopeptidome. The percentage of peptides with basic residues at PΩ was 18.3% (Figure 3b).

### 2.2. HEK-293F Cells Do Not Express HLA-A*03:01 at the Cell Surface

**Figure 4:**
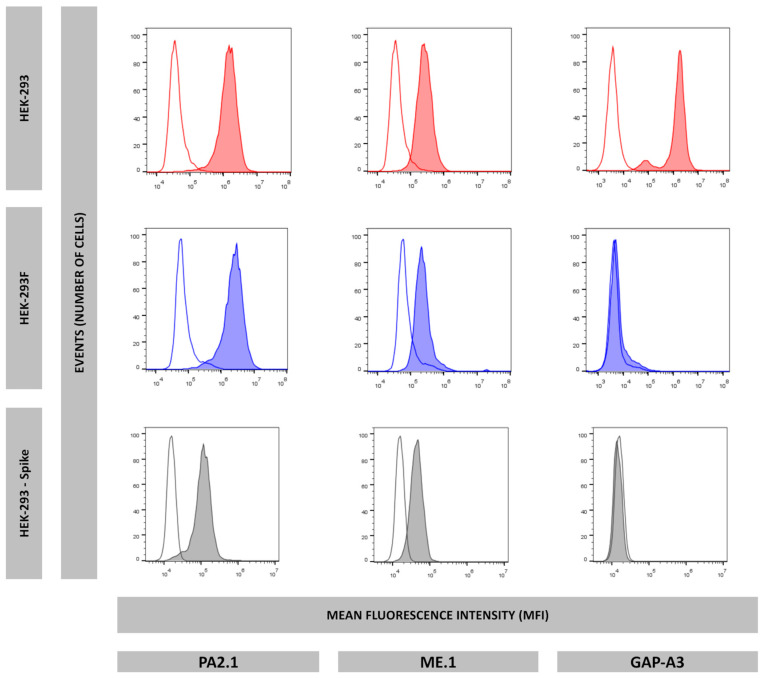
Surface expression of HLA-A2 (PA2.1), HLA-B7 (ME.1), and HLA-A3 (GAP-A3) of HEK-293 (red), HEK-293F (blue), and HEK-293-Spike (grey). Non-colored histograms represent second antibody control.

To explain the lack of HLA-A*03:01 peptide ligands, flow cytometry experiments were performed using specific antibodies recognizing HLA-A2 (PA2.1), HLA-A3 (GAP-A3), and HLA-B7 (ME-1). While all three HLA-I molecules were detected on the cell surface of HEK-293 cells, only HLA-A*02:01 and HLA-B*07:02 were positive in HEK-293F cells, as they showed a negative expression of HLA-A*03:01 (Figure 4).

### 2.3. RT-PCR Shows a Reduction in HLA-A*03:01 and B*07:02 mRNA

**Figure 5:**
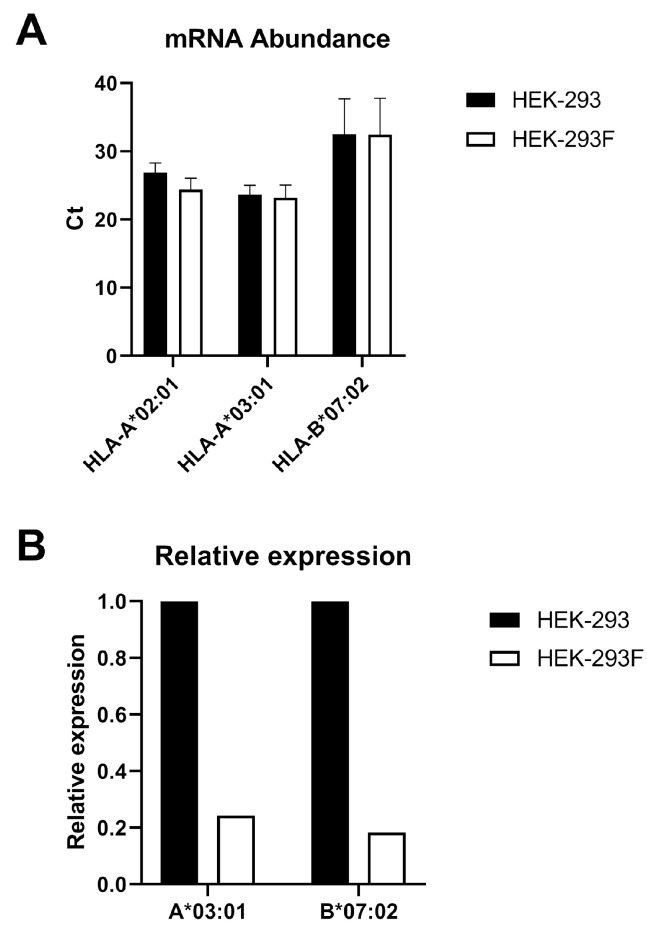
Analysis of the mRNA of HLA-I alleles in HEK-293 and HEK-293F cell lines. (**A**) The different HLA-I alleles were amplified using quantitative PCR (qPCR) from complementary DNA (cDNA) obtained from total messenger RNA (mRNA) extracted from HEK-293 and HEK-293F cells. The Ct value is shown for each allele in both cell types. Error bars represent standard deviation (SD). (**B**). Relative expression of HLA-A*03:01 and HLA-B*07:02 in relation to HLA-A*02:01 expression in the two cell lines. *T*-test analysis showed *p* > 0.05 for both genes.

To investigate whether the absence of HLA-A*03:01 expression occurred at the transcriptional level in HEK-293F cells, total mRNA was purified from HEK-293 and HEK-293F cells and converted into cDNA. Quantitative PCR was then performed using primers specific for HLA-A*02:01, HLA-A*03:01, and HLA-B*07:02. Ct values revealed that HLA-A*03:01 had the highest number of mRNA transcripts (Figure 5A). HLA-A*02:01 was then used as the reference gene to normalize the HLA-A*03:01 and HLA-B*07:02 genes. Figure 5B shows that the HLA-A*03:01/A*02:01 and HLA-B*07:02/A*02:01 ratios were lower in HEK-293F than in HEK-293. Thus, although not significant, relative expression in HEK-293F compared to HEK-293 was 24.3% for HLA-A*03:01 and 18.3% for HLA-B*07:02.

These data indicate that although the relative expression of HLA-A*03:01 decreases in relation to HLA-A*02:01, the same occurs for HLA-B*07:02, which does not explain the absence of HLA-A*03:01 at the cell surface. Therefore, this occurs at the post-transcriptional level.

### 2.4. Absence of HLA-A*03:01 Expression in a HEK-293 Transfectant Expressing the SARS-CoV-2 Spike Protein

Our group is interested in the characterization of peptides derived from the SARS-CoV-2 Spike protein generated by cells expressing different catalytic immunosubunits, including β5i. Thus, the β5i subunit and Spike protein of the Omicron (B.1.1.529) variant genes were cloned in pcDNA3.1 expression vector. First, the β5i construct was transfected into HEK-293 and selected with G418. Cells were cloned using limited dilution, and a derivative cell line expressing β5i and non-expressing β5 was selected (Figure S1). Then, these cells were subsequently transfected with the construct containing the Omicron Spike gene and selected with hygromycin. A transfectant with high surface expression of Spike was selected and from this moment referred to as HEK-293-Spike (Figure S2). About 5 × 10^8^ of HEK-293-Spike cells were lysed, and their HLA-I immunopeptidome was purified and analyzed using mass spectrometry. A total of 996 peptides were identified at 1% FDR (Supplementary Table S3). Following the criteria previously described, a total of 967 unique peptides were identified.

The size distribution of the peptides showed that 653 (67.5%) of them were nonamers, 129 (13.3%) 10-mer, 120 (12.4%) 8-mer, 53 (5.6%) 11-mer, and 12 (1.2%) 12-mer (Figure 2a). Peptides were derived from 800 proteins, mostly located in the nucleus (50.0%) and cytosol (28.3%), with peptides also originating from proteins from plasma membrane (14.8%), secreted (4.2%), mitochondria (2.9%), endoplasmic reticulum (0.8%), and vesicles (0.3%) (Figure 2b).

MHCMotifDecon-1.0 analysis assigned 323 (33.4%) peptides as ligands of HLA-A*02:01, 566 (58.5%) peptides bound to HLA-B*07:02, and 58 (6.0%) to HLA-C*07:02 (Figure 3a). Eleven (1.1%) peptides were considered as trash and were not assigned to any allotype. Surprisingly, only 9 (0.9%) peptides were predicted to bind to HLA-A*03:01 (Figure 3a). The percentage of peptides with basic residues at PΩ was 1.9% (Figure 3b).

Flow cytometry analysis showed that HLA-A*03:01 was not expressed in this transfectant (Figure 4). Thus, HLA-A*03:01 expression was lost during Spike gene transfection, suggesting that HLA-A*03:01 expression is specifically unstable in HEK-293 cells.

### 2.5. Theoretical Binding Affinity of the Peptides Assigned to Each HLA-I Allotype

**Figure 6:**
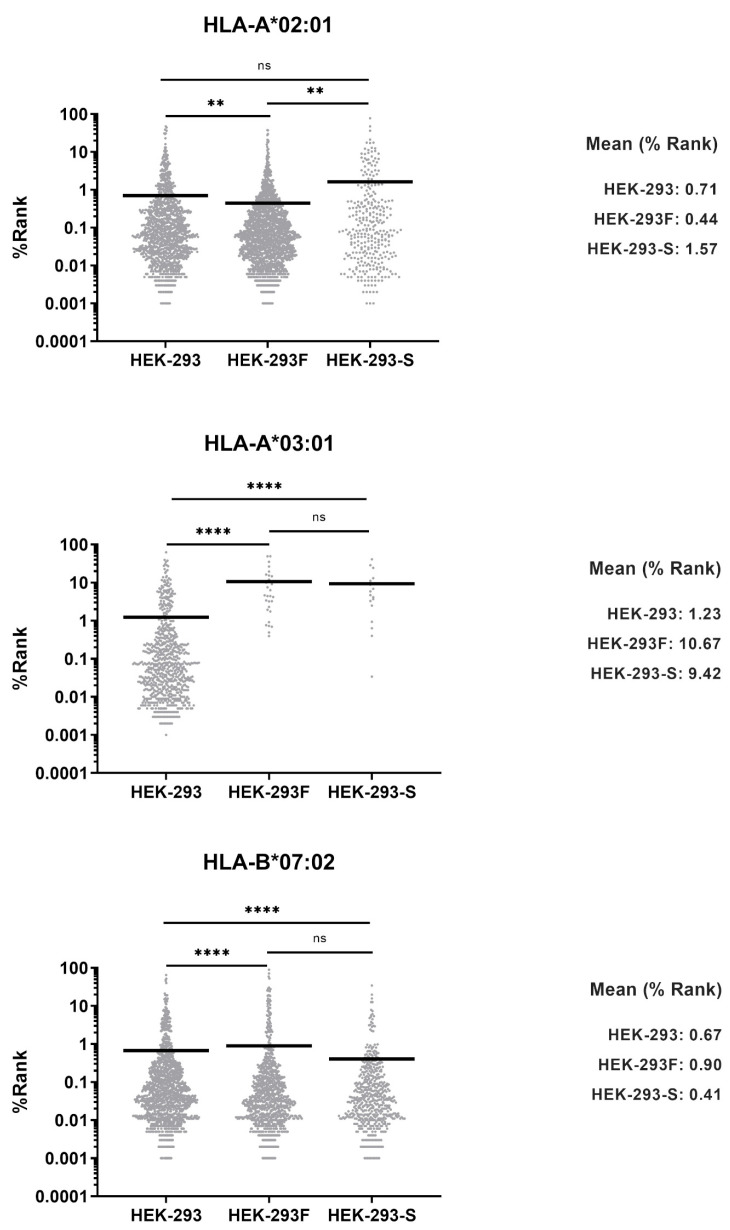

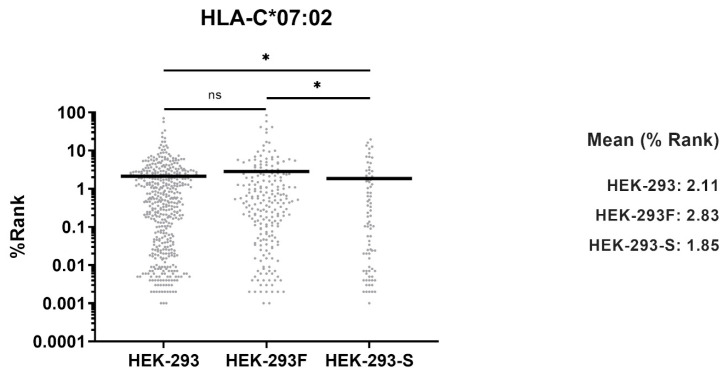
Theoretical affinity of the peptides assigned to each HLA-I allotype. Theoretical affinity was calculated using NetMHCpan 4.1 software. Each peptide was assigned to a given allotype according to theoretical affinity (lowest %Rank). Symbols represent individual peptides, and horizontal lines represent the median. * = 0.01 < *p* < 0.1; ** = 0.001 < *p* < 0.01; **** = *p* < 0.0001; ns = not significant based on Mann–Whitney U test. Theoretical mean is also shown.

Peptide binding affinity to molecules they were assigned to bind was calculated using NetMHCpan-4.1 software (Supplementary Table S4). Binding affinities values were compared by pairs without multiple testing correction. Although statistical differences were detected in multiple pairs, these were due to the high number of peptides. However, affinity was similar in all cell lines for -A*02:01, -B*07:02, and -C*07:02 but it was lower in HEK-293F for -A*03:01 (Figure 6). This is probably because of the low number of peptides bound to this molecule, some of which may be contaminants, as their anchor motifs do not correspond to any HLA-I molecule present in the cells.

## 3. Discussion

The HEK-293 cell line and its derivatives have been commonly employed, as they are highly transfectable. Multiple variants of this cell line have been generated. HEK-293 and HEK-293T have been commonly used for protein expression and purification. In addition, HEK-293T have been used to identify peptides derived from SARS-CoV-2 proteins presented by HLA-I [8,10].

The detection of scarce peptides presented by HLA-I molecules may require large cell quantities to increase the possibility to detect them. Suspension-growing cells are ideal to obtain high density numbers in a relatively short time. The HEK-293F cell line has the potential to be a suitable model comparable to HEK-293 in antigen processing and presentation.

HLA typing of HEK-293T cells has been published, but results are contradictory: some studies report that HEK-293T cells are homozygous for HLA-A*02:01 [10], while other report homozygosity for HLA-A*03:01 [11]. In our analysis, HLA typing of HEK-293 and HEK-293F cell lines showed that HLA-A*03:01 allele is present alongside HLA-A*02:01, HLA-B*07:02, and HLA-C*07:02. Therefore, the absence of HLA-A*03:01 gene is not responsible for the lack of expression of this allotype.

In this work, HEK-293F cells were used to analyze the HLA-I immunopeptidome. Analysis exhibited an absence of HLA-A*03:01 peptide ligands, which, to our knowledge, has not been reported previously. Analysis of HEK-293 HLA-I immunopeptidome confirmed that the parental cell line expresses all HLA-I allotypes on the cell surface.

In addition, our group generated several HEK-293-derived cell transfectants expressing different catalytic subunits of the 20S proteasome together with SARS-CoV-2 Spike protein. One of them spontaneously lost the expression of the allotype HLA-A*03:01, while other transfectants maintained the expression of all HLA-I molecules, confirming that HEK-293 lose HLA-A*03:01 expression while maintaining the expression of the rest of HLA-I molecules. The process of adapting a cell line to grow in suspension differs from the generation of a transfectant cell line expressing a specific protein. Nevertheless, both HEK-293F and HEK-293-Spike cells lacked only HLA-A*03:01. This strongly suggests that this cell line tends to specifically lose the expression of this allotype.

The use of a cell line as professional antigen-presenting cells (APCs) requires the expression of co-stimulatory molecules. Recently, a cellular model using HEK-293 cells has been established by transfecting co-stimulatory molecules (CD80, CD83, CD137L) to assess CD8+ T cell activation efficiency against epitopes presented by HLA-A*03:01 [12]. Our data show that HEK-293F cells cannot be employed as APCs similarly to HEK-293 cells for peptides presented in the context of HLA-A*03:01, as those peptides will not be presented by HEK-293F.

As said above, according to the literature, HEK-293T cells HEK-293T cells express the same HLA-I antigens at the cell surface as those observed in this work for HEK-293F. Thus, in studies in which HEK-293T cells were used for the characterization of HLA-I SARS-CoV-2-derived peptide ligands, those presented by HLA-A*03:01 were not detected in the immunopeptidomes.

Thus, in the case HEK-293 where transfectants are used either as a source of HLA-I ligands or as APCs for antigen presentation, the expression of HLA-A*03:01 must be monitored, as this allotype’s expression can be spontaneously lost during transfection and selection.

The mechanism through which HLA-A*03:01 is specifically absent is not easy to explain. It is well-known that tumor and transformed cells can lose HLA-I expression during immune responses, and, in some cases, specific allotypes can be lost, while others are maintained. It is possible that a specific HLA-I molecule presents an immunogenic peptide and the loss of this allotype impairs the immune response against this peptide–MHC complex, while the expression of the remaining HLA-I molecules avoid NK activation, being an immune escape mechanism. However, in the case of HEK-293 cells, no pressure is exerted during the generation of transfectant cell lines, and other mechanisms may be involved.

In many cases, transformed cells are genetically unstable. For instance, HEK-293 cells have been reported to present genomic instability under selective conditions [6]. As HLA-I genes are in the class I region of the MHC and very close in the genome, the mechanism by which the expression of *HLA-A03:01* is so easily lost is not clear.

## 4. Materials and Methods

### 4.1. Cell Lines, HLA Typing, and Antibodies

The Human Embryonic Kidney (HEK)-293 cell line is a human cell line of embryonic origin immortalized by transduction with adenovirus 5 [2]. Cells were cultured in D-MEM (Gibco—ThermoFisher, Waltham, MA, USA) supplemented with 10% FBS (Gibco—ThermoFisher, Waltham, MA, USA) and cultured at 37 °C with 5% CO_2_.

HEK-293F is a variant of HEK-293 cells adapted to grow in suspension. Cells were grown in FreeStyle 293 medium (Gibco—ThermoFisher, Waltham, MA, USA) at 37 °C with 8% CO_2_ and orbital shaking 150 rpm.

The gDNA extraction and HLA typing were performed as previously described [13,14].

The following mAbs were used: W6/32 (IgG2a, specific for a monomorphic HLA-A,B,C determinant), HC10 (IgG2a, specific for denatured and other forms of HLA class I heavy chain not associated to β2m), PA2.1 (IgG1, specific for an epitope on the alpha 2 domain of HLA-A2), ME1 (IgG1, specific for HLA-B27, -B7, -B22), GAP-A3 (IgG2a specific for HLA-A3) (BD Biosciences, San Jose, CA, USA, Ref. 566605). Hybridoma of all antibodies were grown in the laboratory, except GAP-A3.

### 4.2. Flow Cytometry

Flow cytometry analysis was performed as previously described [15]. Approximately 2 × 10^5^ HEK-293, HEK-293F, or HEK-293-Spike transfectant cells were washed twice in 200 μL of PBS 2%FBS and resuspended in 50 μL of undiluted mAb supernatant (anti-HLA-A3 was diluted 1:200 in PBS 2%FBS). After incubating for 30 min, cells were washed three times in 200 μL of PBS 2%FBS and resuspended in 50 μL of fluorescein isothiocyanate-conjugated anti-mouse IgG rabbit antiserum (Calbiochem-Novabiochem GmbH, Schwalbach, Germany) diluted 1:200, incubated for 30 min, and washed three times in 200 μL of PBS. All operations were performed at 4 °C. Flow cytometry was conducted using a CytoFlex Cytometer (Beckman Coulter Life Sciences, Indianapolis, IN, USA).

### 4.3. Isolation of the HLA Class I-Bound Peptide Pool

Immunoprecipitation of MHC class I molecules and subsequent isolation of their associated peptidomes was performed as previously described [14,16,17] starting from pellets of 5 × 10^8^ cells. In brief, cells were lysed in Lysis buffer (LB: 20 mM Tris, 150 mM NaCl, pH 7.5) containing 1% Igepal CA-630 (Sigma-Aldrich, St. Louis, MO, USA, Ref. I8896) and a protease inhibitors cocktail (cOmplete, Roche Applied Science, Mannheim, Germany, Ref. 11697498001). The lysate was then centrifuged for 10 min at 2500× *g*, and 1 h at 100,000× *g*. Afterwards, the supernatant was subjected to affinity chromatography using the W6/32 mAb coupled to CNBr-activated sepharose beads (GE Healthcare, Little Chalfont, UK, Ref. 17043001).

MHC class I-bound peptides were eluted with 0.1% TFA, and peptides were isolated from α chain and β2m through ultrafiltration through a 2 kDa filter. The peptide-containing fractions were combined and concentrated in a SpeedVac, desalted, dried to completeness, and redissolved in 0.1% formic acid prior to LC-MS analysis.

### 4.4. LC-MS/MS Analysis

Peptides were dissolved in 0.1% formic acid for LC-MS/MS analysis.

For HEK-293 and HEK-293-Spike immunopeptidomes, MS analysis was performed using an Ultimate 3000 HPLC (Thermo Fisher Scientific, Waltham, MA, USA) coupled online to an Orbitrap Exploris 240 mass spectrometer (Thermo Fisher Scientific, Waltham, MA, USA). The HPLC was equipped with a PepMap Neo C18 trapping column (300 µm × 5 mm; Thermo) and a PepMap RSLC C18 column (75 µm × 50 cm; Thermo Fisher Scientific, Waltham, MA, USA). Solvents A and B were, respectively, 0.1% formic acid and 0.1% formic acid, 80% acetonitrile. The chromatography was performed at 50 °C at a flowrate of 250 nL/min. The following gradient was employed: 4% B for 3 min, linear increase to 35% B in 120 min, a linear increase to 90% B in 1 min and 90% B for 5 min. The mass spectrometer was operated in DDA mode. Each acquisition cycle consisted of a survey scan (350–1200 m/z) at 60,000 resolution (FWHM) and up to 25 MS/MS scans at 15,000 resolution (FWHM). Peptides with charges 1 to 5 were selected for HCD fragmentation with an applied HCD collision energy of 30%. A dynamic exclusion window of 25 s was applied after the first fragmentation of each precursor ion.

In the case of HEK-293F immunopeptidome, the LC-MS/MS analysis was performed using a Vanquish Neo HPLC (Thermo Fisher Scientific, Waltham, MA, USA) and an Orbitrap Eclipse mass spectrometer (Thermo Fisher Scientific, Waltham, MA, USA). The HPLC setup included a µPac trapping column (Thermo Fisher Scientific, Waltham, MA, USA) and µPac Neo column (50 cm; Thermo Fisher Scientific, Waltham, MA, USA). Solvent A was 0.1% formic acid, and solvent B was 0.1% formic acid 80% acetonitrile. The separation was performed at 60 °C at a flowrate of 250 nL/min under the following gradient elution conditions: linear increase from 4% to 30% B in 75 min, linear increase to 50% B in 5 min, a linear increase to 90% B in 5 min and 90% B for 10 min. Data were acquired in DDA mode. Each acquisition cycle had a maximum duration of 3 s and included an MS scan (300–1400 m/z) at 120,000 resolution (FWHM) followed by MS/MS scans at 30,000 resolution (FWHM). Both MS and MS/MS spectra were acquired in the orbitrap analyzer. Peptides with charges 1 to 4 were selected for HCD fragmentation with an HCD collision energy of 30%. A dynamic exclusion window of 10 s was applied after the first fragmentation of each precursor ion.

### 4.5. MS/MS Ions Search, Peptide Identification, and Binding Motif Deconvolution

Raw files were searched with a Deep novo peptidome workflow in PEAKS Studio 12.0 (Bioinformatics Solutions Inc., Waterloo, ON, Canada) against the human reference proteome (20,413 entries), downloaded from Uniprot on 11 January 2024. When appropriate, the sequence of the spike protein of SARS-CoV-2 (Omicron strain) was included in the database. For data refinement, the options “Mass Correction” and “Associate feature with Chimera” were enabled. No enzyme was selected, and MS and MS/MS tolerances were set at 10 ppm and 0.02 Da, respectively. The following variable modifications were considered: oxidation of M and pyroglutamic acid formation from N-terminal Q. Results were filtered at an FDR < 1% at the peptide level, and only the identifications by database search (de novo results excluded) were considered.

### 4.6. Subcellular Location Assignation

Protein location was determined using Uniprot database. For those proteins in more than one compartment, the most common according to its biological function was assigned.

### 4.7. In Silico Theoretical Binding Allele Assignation

Assignation of each peptide as a ligand of HLA-A*02:01, HLA-A*03:01, HLA-B*07:01, or HLA-C*07:01 was performed with unique peptides of 8 to 12 residues long from HEK-293, HEK-293F, or HEK-293-Spike cells using MHCMotifDecon-1.0 [18] (URL accessed on 19 November 2025, https://services.healthtech.dtu.dk/services/MHCMotifDecon-1.0/).

### 4.8. qPCR of HLA-I Alleles

RNA extraction. mRNA was extracted from dry pellets containing 10^7^ cells using a RNeasy Plus Mini Kit (QIAGEN, Hilden, Germany) following the manufacturer’s instructions. The RNA concentration was measured using a Nanodrop spectrophotometer (Thermo Fisher Scientific, Waltham, MA, USA).

RT-PCR. cDNA synthesis was carried out using Moloney Murine Leukemia Virus Reverse Transcriptase (M-MLV RT) (Invitrogen, Carlsbad, CA, USA) from the previously purified RNA. First, a mixture containing Oligo(dT)s (1 μg/μL), dNTPs (10 mM) and DEPC-treated water was prepared, to which the RNA sample was added. This mixture was then incubated at 65 °C for 5 min in a SimpliAmp Thermal Cycler (Thermo Fisher Scientific, Waltham, MA, USA). A second mixture containing 5× buffer, DTT (0.1 M), and RNaseOUT (40 U/μL) was then added, after which the mixture was incubated for 2 min at 37 °C. Finally, M-MLV reverse transcriptase was added, and the reaction was incubated for 50 min at 37 °C, followed by 15 min at 70 °C in the SimpliAmp Thermal Cycler (Thermo Fisher Scientific, Waltham, MA, USA). The concentration of the resulting cDNA was determined using a Nanodrop spectrophotometer (Thermo Fisher Scientific, Waltham, MA, USA).

qPCR. The genomic sequences of the HLA-A*02:01, HLA-A*03:01, and HLA-B*07:02 alleles were obtained from the GenBank database. A BLAST comparison of HLA-A*02:01 and HLA-A*03:01 identified a polymorphic region (bases 62–132) containing nine mismatches. This region was used to design allele-specific primers using the NCBI Primer Design Tool.

cDNA, synthesized by reverse transcribing the target RNA, was amplified using SsoAdvanced Universal SYBR Green Supermix (Bio-Rad Laboratories, Hercules, CA, USA) and the following allele-specific primers:

HLA-A*02:01: Forward: 5′-CGCATATGACTCACCACGCT-3′ Reverse: 5′-AGGTCAGTGTGATCTCCGA-3′; HLA-A*03:01: Forward: 5′-CACATATGACCCACCACCCC-3′. Reverse: 5′-GCCAGGTCGATCTCCG-3′; HLA-B*07:02: forward: 5′-GCTCCCACTCCGAAAGGTATTTCT-3′. Reverse: 5′-TCTCTCGGTCGATCTGTGCCTG-3′ in 96-well plates (Bio-Rad). Reactions were run on a CFX Opus thermocycler (Bio-Rad Laboratories, Hercules, CA, USA) under RT-PCR conditions (95 °C for 30 s, followed by 40 cycles of 95 °C for 5 s and 65 °C for 30 s). All experiments were performed in triplicate on three independent days.

### 4.9. In Silico Prediction of MHC Binding Affinities

Theoretical binding affinities for HLA-A*02:01, HLA-A*03:01, HLA-B*07:02, and HLA-C*07:02 were calculated independently for ligands of 8 to 12 residues long using the NetMHCpan4.1 server [19] (URL accessed on 19 November 2025, https://services.healthtech.dtu.dk/service.php?NetMHCpan-4.1).

### 4.10. Western Blot

Dry pellets of about 10^7^ HEK-293 and HEK-293β5i cells were lysed in Lysis buffer (20 mM Tris-HCl, 150 mM NaCl, 1% NP40 and protease inhibitors Roche). Protein concentration was quantified (BCA Protein Assay (Thermo Fisher Scientific, Waltham, MA, USA)) using a Viktor3 (PerkinElmer, Waltham, MA, USA). About 20 μg of protein were loaded and separated using SDS-PAGE electrophoresis in a 14% acrylamide gel. Proteins were transferred to a Poliviliden Fluorur (PVDF) membrane (Bio-Rad Laboratories, Hercules, CA, USA) at 300 mA for one hour. Membranes were then blocked for one hour with T-PBS (PBS, 0.1% Tween 20 (PanReac AppliChem, Barcelona, Spain), 5% skimmed milk). After four washes with T-PBS, membranes were incubated with their respective antibody diluted in T-PBS for one hour at RT, or overnight at 4 °C. Finally, membranes were incubated with the secondary antibody diluted 1/10,000 in T-PBS together with the Precision Plus Protein (Bio-Rad), both conjugated with HRP, for one hour at RT. Membranes were imaged using the Clarity Western ECL Substrate kit (Bio-Rad) with the Versadoc ImagingSystem (Bio-Rad Laboratories, Hercules, CA, USA) and the QuantityOne 4.6 software (Bio-Rad Laboratories, Hercules, CA, USA). Images were analyzed with ImageJ software (v1.47, National Institutes of Health, Bethesda, MD, USA).

### 4.11. Statistical Analysis

Differences in MHC binding affinities were analyzed using the Mann–Whitney U test. Different *p* values were considered.

Differences in Ct values for mRNA expression of HLA-I alleles were analyzed using a *t*-test. A *p*-value of 0.05 was considered significant.

## 5. Conclusions

1. HEK-293 cells tend to specifically lose the HLA-I allotype HLA-A*03:01.
2. HEK-293F cells cannot be employed as comparable to HEK-293 cells as a source of HLA-I peptide ligands or as antigen-presenting cells, as they do not express HLA-A*03:01.
3. HLA-A*03:01 expression should be evaluated with specific antibodies after gene transfection or cell selection.

## Data Availability

The original contributions presented in this study are included in the article/Supplementary Material. Further inquiries can be directed to the corresponding author(s).

## Supplementary Materials

The following supporting information can be downloaded at: https://www.mdpi.com/article/10.3390/ijms262311357/s1.

## Abbreviations

The following abbreviations are used in this manuscript:

HEK	Human Embryonic Kidney
HLA	Human Leukocyte Antigen
pHLA	Peptide–HLA complex
FDR	False Discovery Rate
