# Supplementary material for: Specific Instability of HLA-A*03:01 Expression in HEK-293 Cells
Source: Int J Mol Sci. 2025 Nov 24;26(23):11357. doi: 10.3390/ijms262311357 (PMC12692271; doi:10.3390/ijms262311357)

Experiment 1a

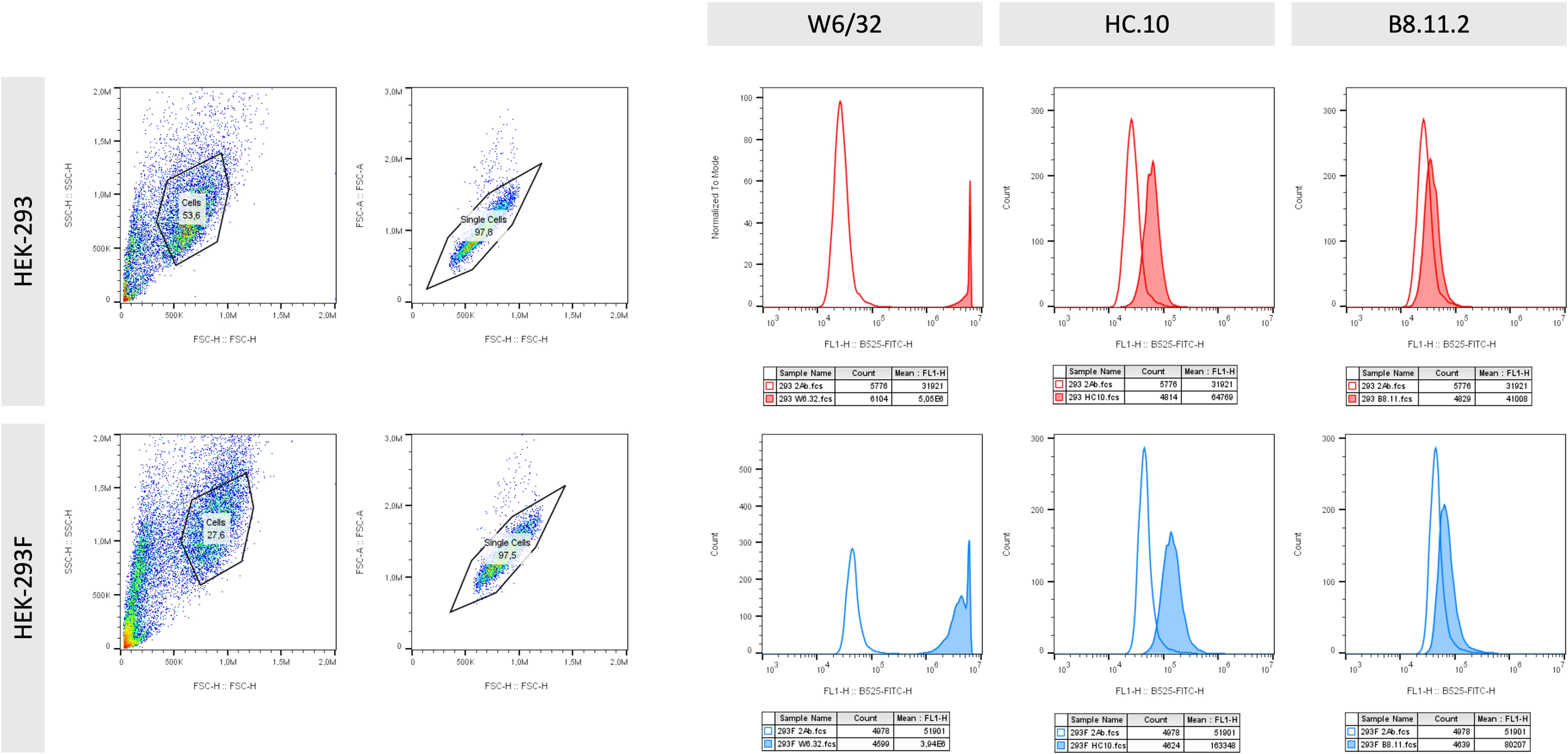

Experiment 1b

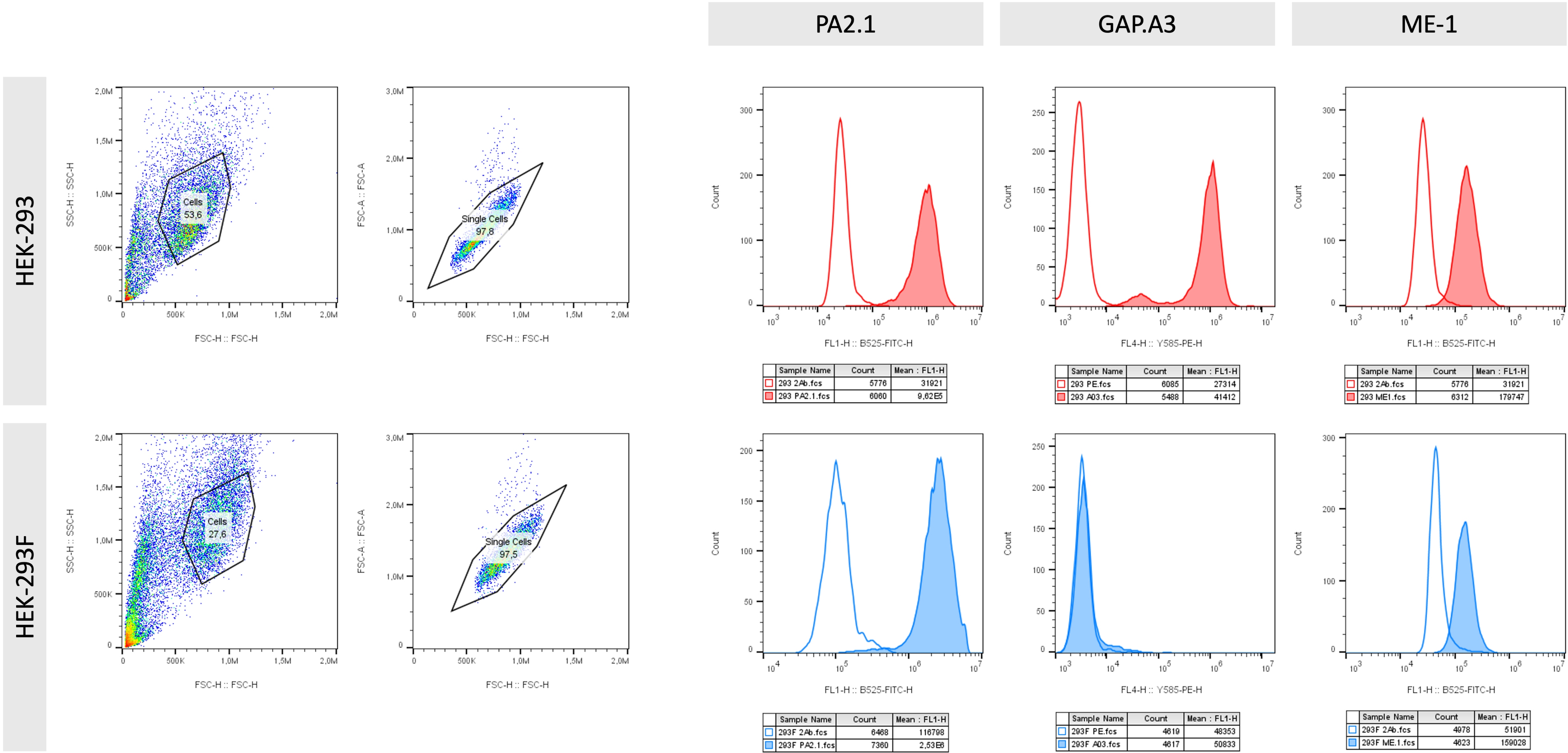

Experiment 2a

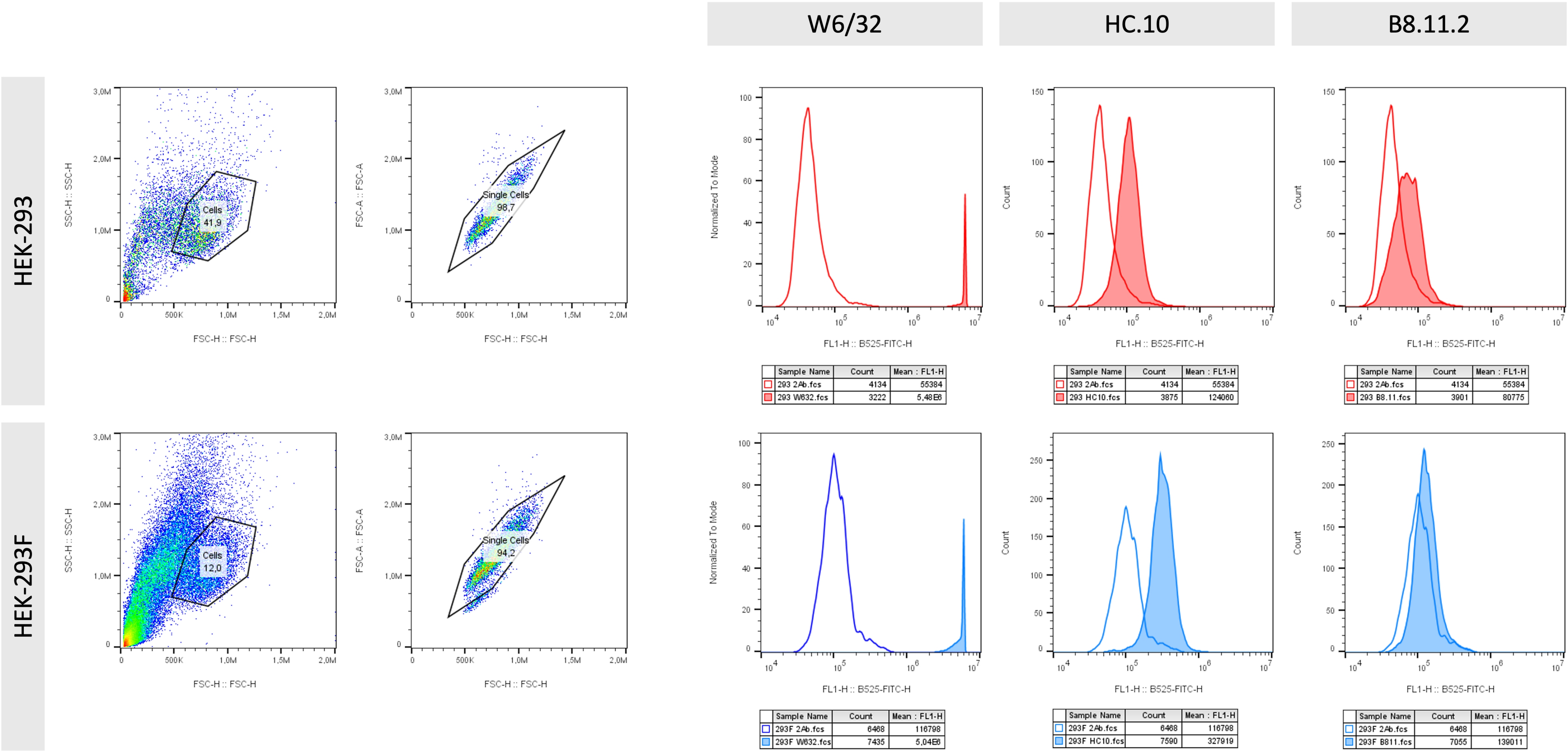

<u>Experiment 2b</u>

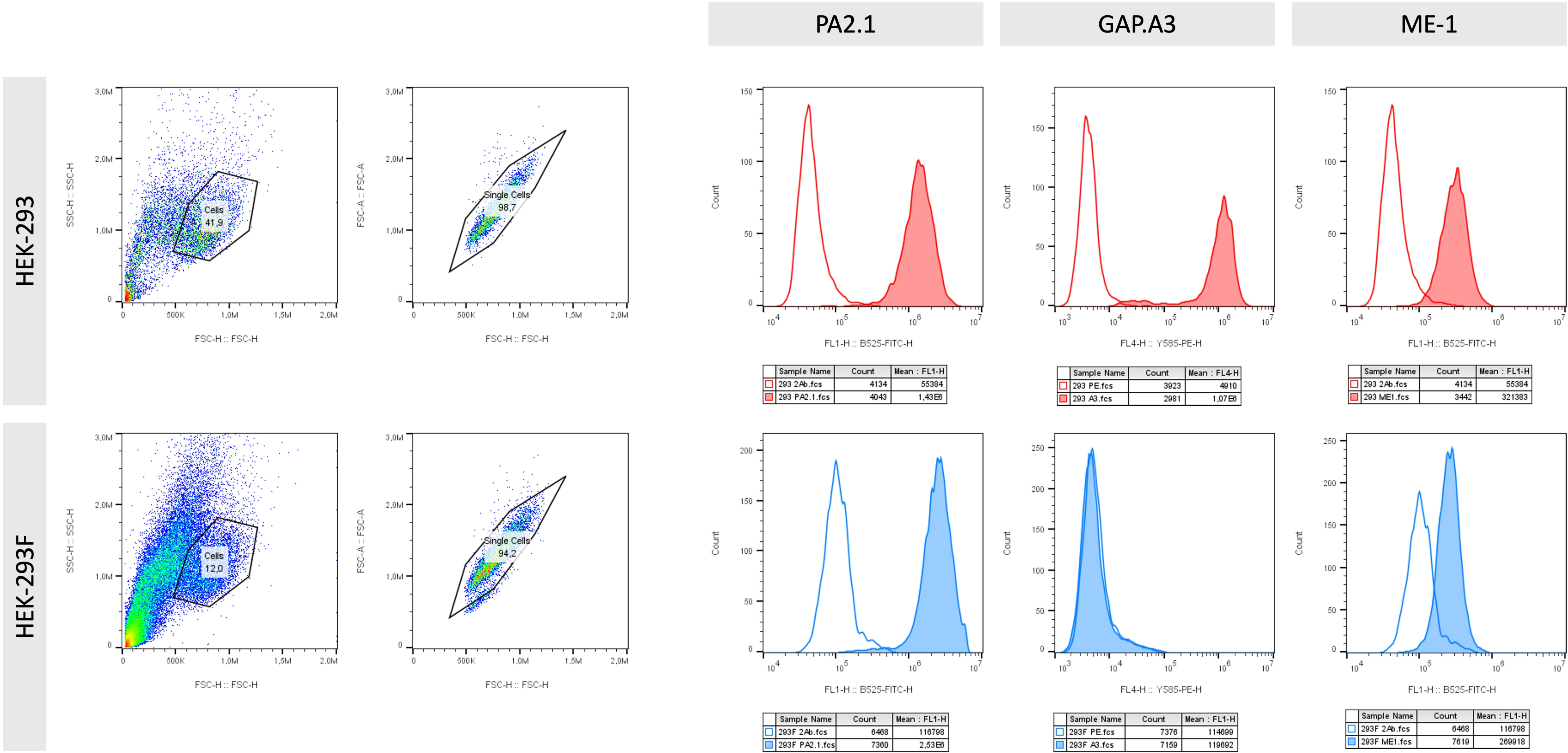

Sample Name	Count	Mean : FL1-H
293 2Ab.fcs	4134	55384
293 PA2.1.fcs	4043	1,43E6

Sample Name	Count	Mean : FL4-H
293 PE.fcs	3923	4910
293 A3.fcs	2981	1,07E6

Sample Name	Count	Mean : FL1-H
293 2Ab.fcs	4134	55384
293 ME1.fcs	3442	321383

Sample Name	Count	Mean : FL1-H
293F 2Ab.fcs	6468	116798
293F PA2.1.fcs	7360	2,53E6

Sample Name	Count	Mean : FL1-H
293F PE.fcs	7376	114699
293F A3.fcs	7159	119692

Sample Name	Count	Mean : FL1-H
293F 2Ab.fcs	6468	116798
293F ME1.fcs	7619	269918

Experiment 3 (293)

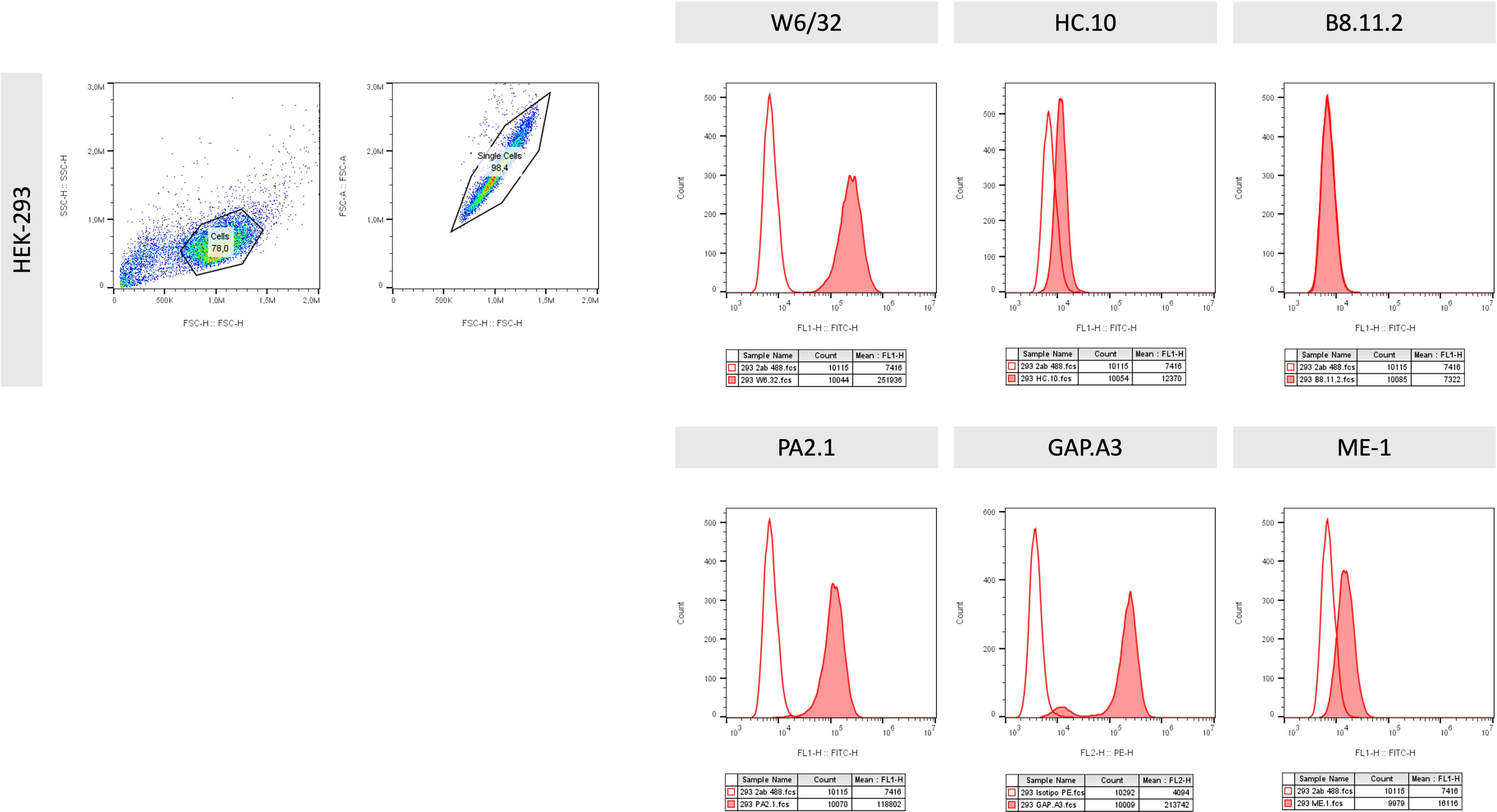

Experiment 3 (293-Spike)

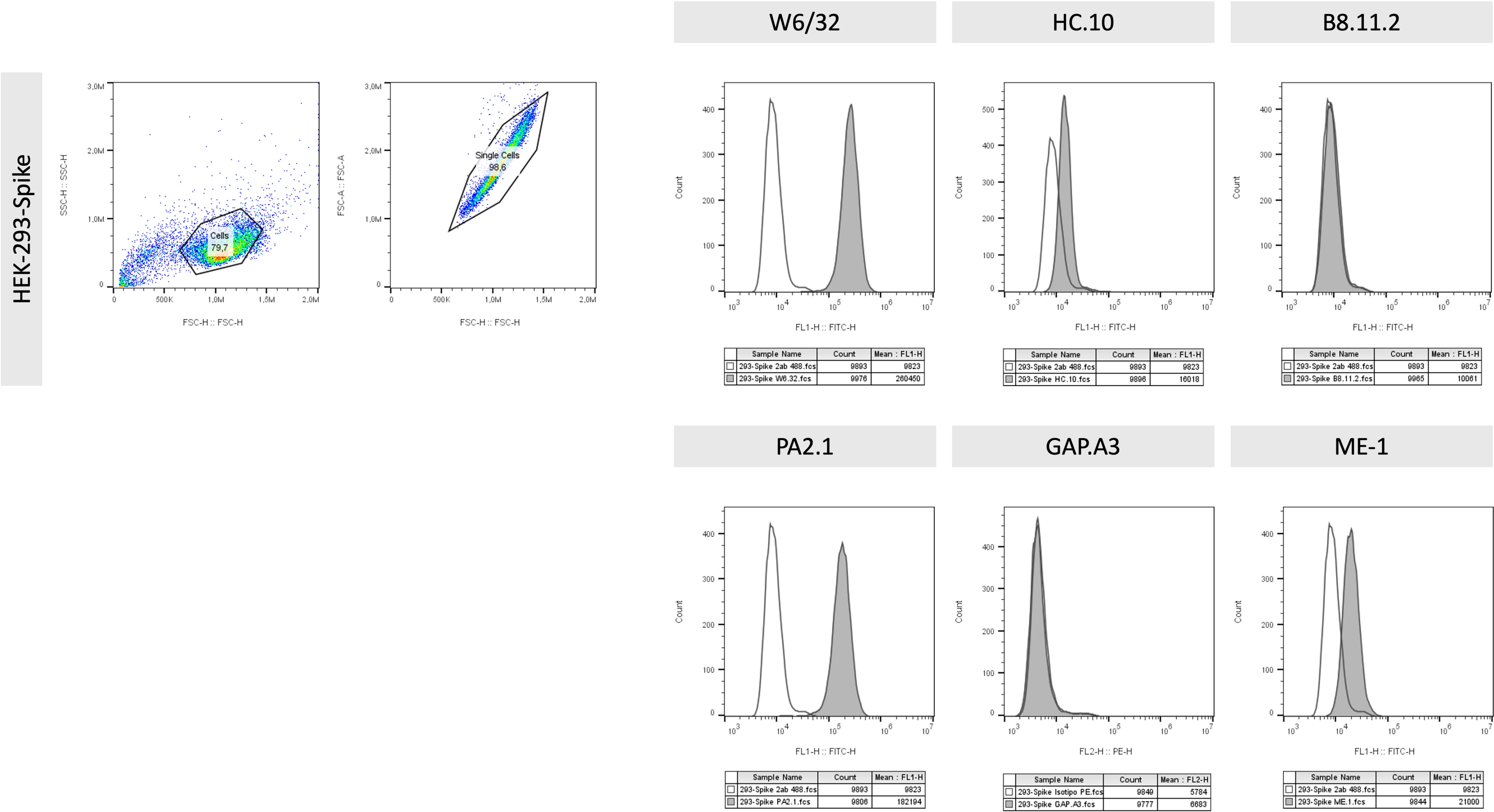

Experiment 4 (293-Spike)

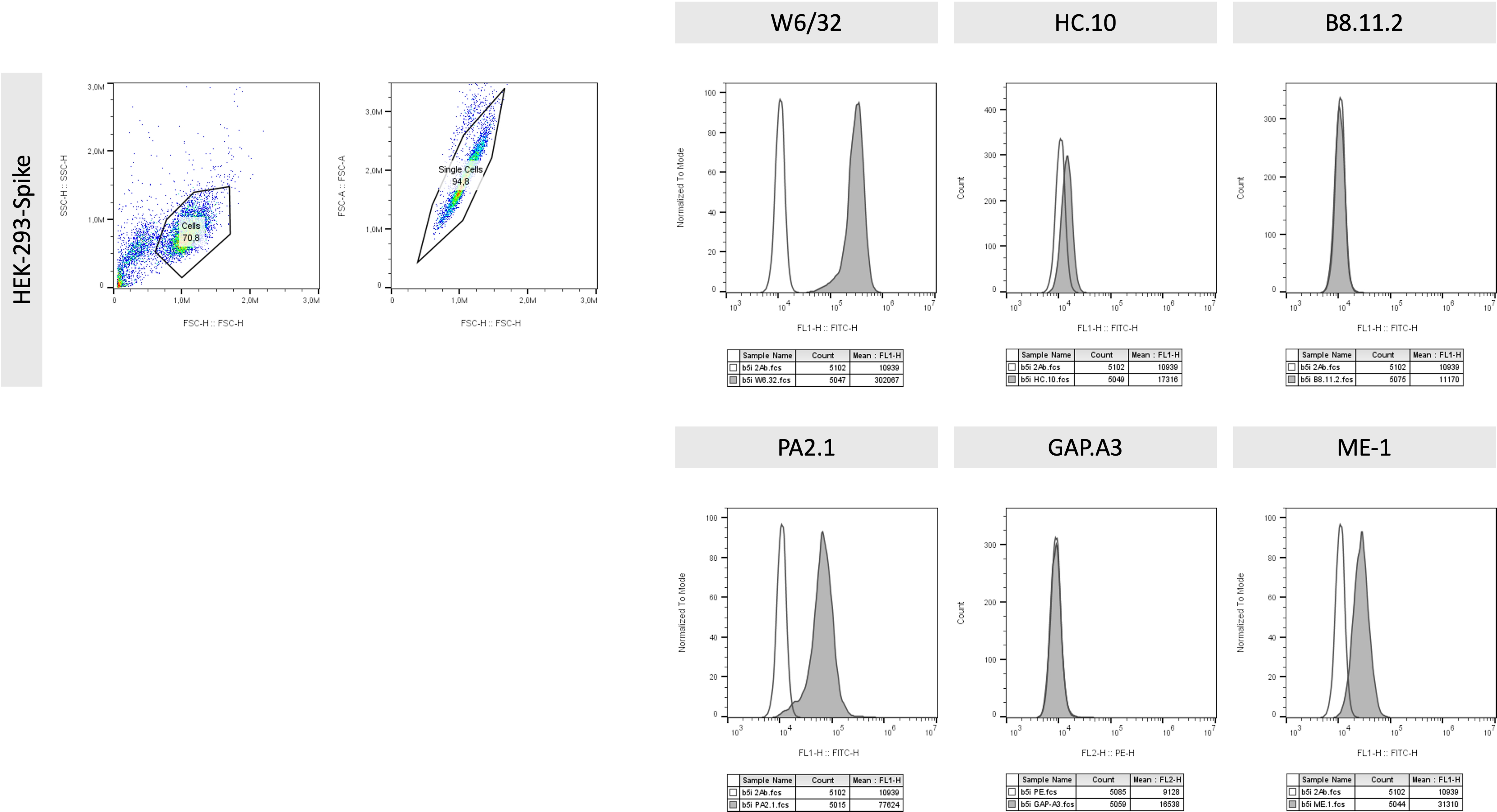

Supplement: Supplementary file 1 [file ijms-26-11357-s001.zip › Flow cytometry additional experiments.pdf]
