# Supplementary figures and images for: Specific Instability of HLA-A*03:01 Expression in HEK-293 Cells
Source: Int J Mol Sci. 2025 Nov 24;26(23):11357. doi: 10.3390/ijms262311357 (PMC12692271; doi:10.3390/ijms262311357)

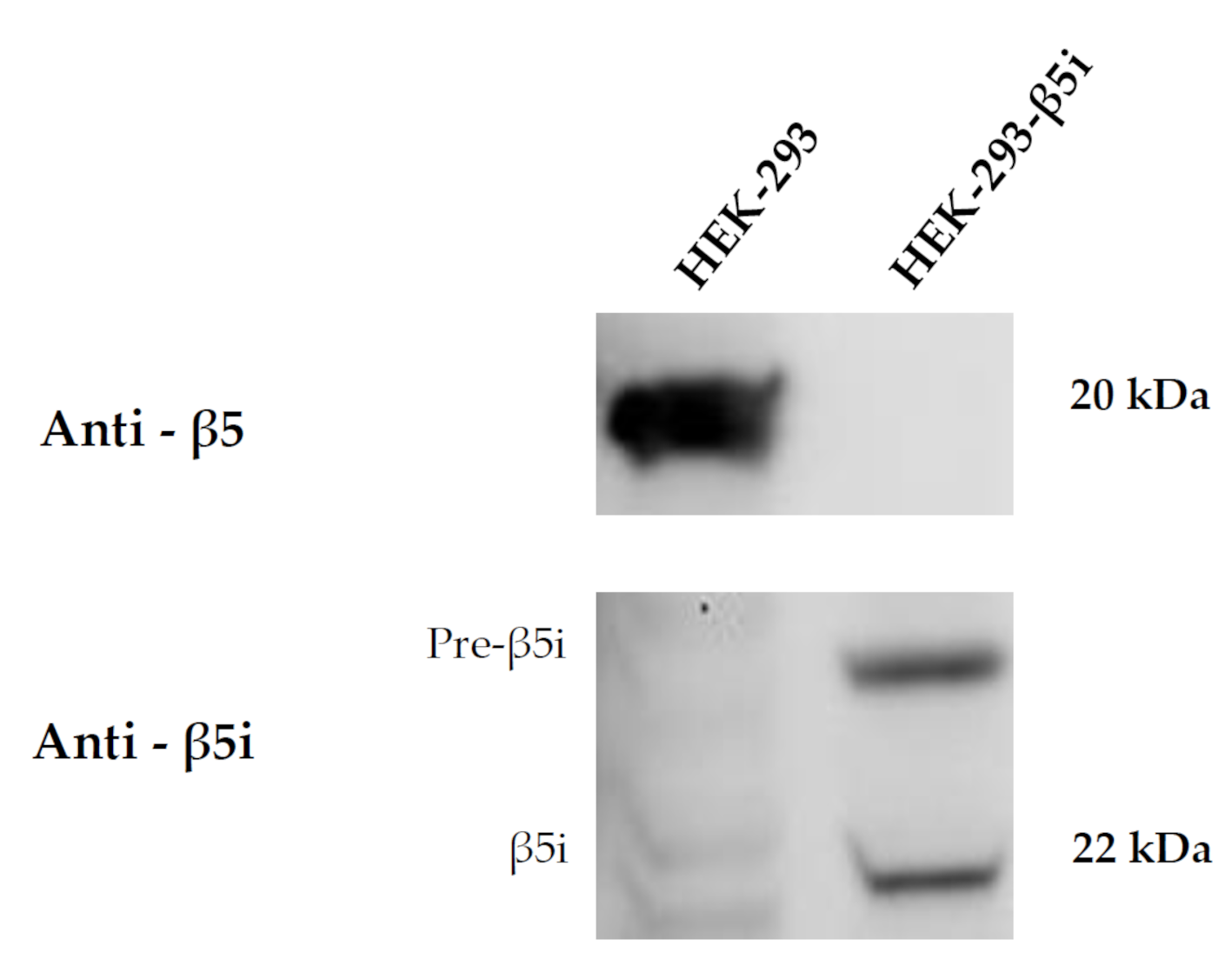

Supplement: Supplementary file 1 [file ijms-26-11357-s001.zip › Area et al Figure S1.tif]

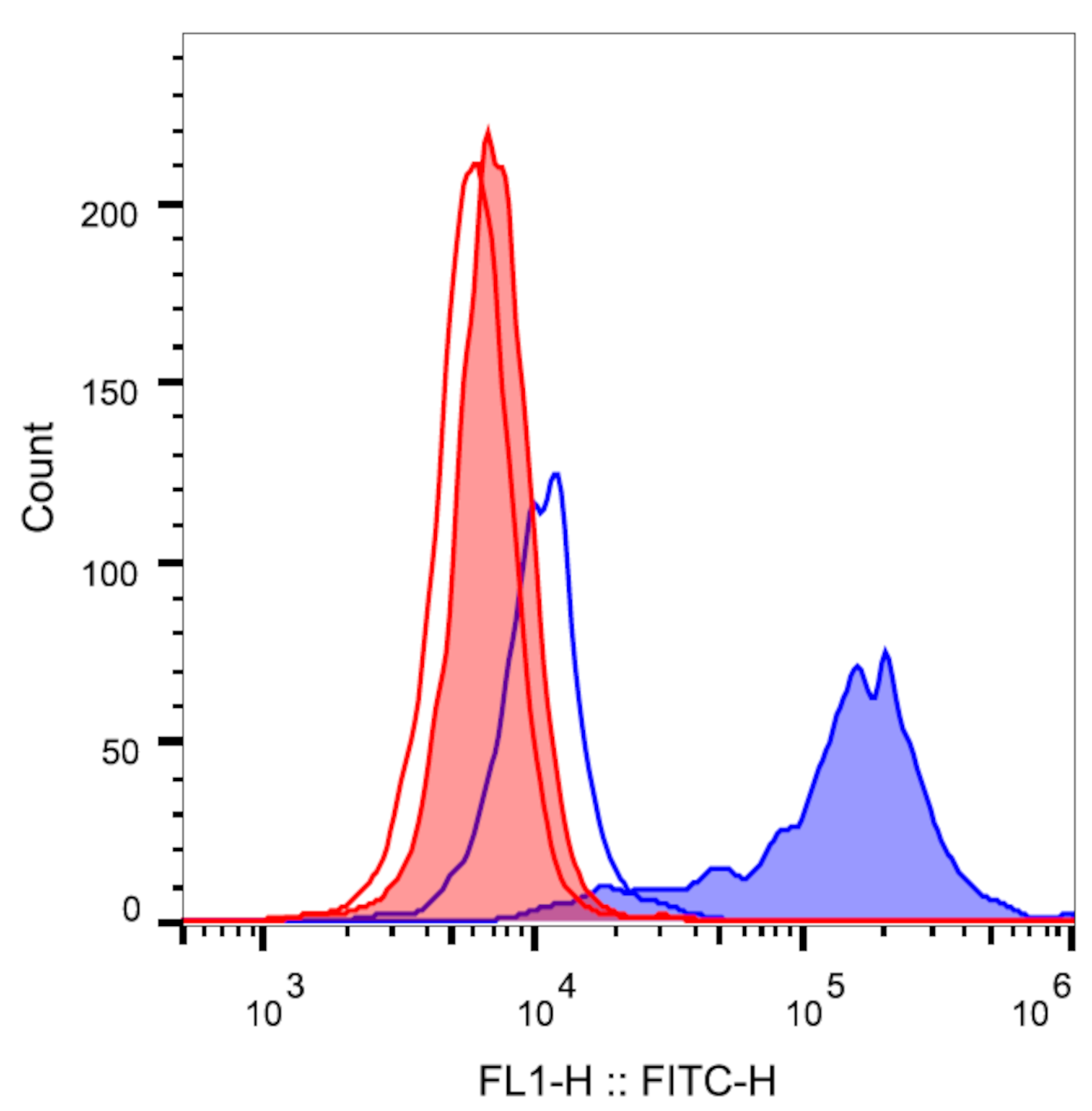

Supplement: Supplementary file 1 [file ijms-26-11357-s001.zip › Area et al Figure S2.tif]
